# *Plasmodium falciparum* ookinete expression of plasmepsin VII and plasmepsin X

**DOI:** 10.1186/s12936-016-1161-5

**Published:** 2016-02-24

**Authors:** Fengwu Li, Viengngeun Bounkeua, Kenneth Pettersen, Joseph M. Vinetz

**Affiliations:** Division of Infectious Diseases, Department of Medicine, University of California San Diego, 9500 Gilman Drive, La Jolla, CA 92093-0760 USA

**Keywords:** *Plasmodium falciparum*, Plasmepsin, Aspartic protease, Transmission-blocking, Gene expression, Transcriptomics, Cell biology, Biochemistry

## Abstract

**Background:**

*Plasmodium* invasion of the mosquito midgut is a population bottleneck in the parasite lifecycle. Interference with molecular mechanisms by which the ookinete invades the mosquito midgut is one potential approach to developing malaria transmission-blocking strategies. *Plasmodium* aspartic proteases are one such class of potential targets: plasmepsin IV (known to be present in the asexual stage food vacuole) was previously shown to be involved in *Plasmodium gallinaceum* infection of the mosquito midgut, and plasmepsins VII and plasmepsin X (not known to be present in the asexual stage food vacuole) are upregulated in *Plasmodium falciparum* mosquito stages. These (and other) parasite-derived enzymes that play essential roles during ookinete midgut invasion are prime candidates for transmission-blocking vaccines.

**Methods:**

Reverse transcriptase PCR (RT-PCR) was used to determine timing of *P. falciparum* plasmepsin VII (PfPM VII) and plasmepsin X (PfPM X) mRNA transcripts in parasite mosquito midgut stages. Protein expression was confirmed by western immunoblot and immunofluorescence assays (IFA) using anti-peptide monoclonal antibodies (mAbs) against immunogenic regions of PfPM VII and PfPM X. These antibodies were also used in standard membrane feeding assays (SMFA) to determine whether inhibition of these proteases would affect parasite transmission to mosquitoes. The Mann–Whitney U test was used to analyse mosquito transmission assay results.

**Results:**

RT-PCR, western immunoblot and immunofluorescence assay confirmed expression of PfPM VII and PfPM X in mosquito stages. Whereas PfPM VII was expressed in zygotes and ookinetes, PfPM X was expressed in gametes, zygotes, and ookinetes. Antibodies against PfPM VII and PfPM X decreased *P. falciparum* invasion of the mosquito midgut when used at high concentrations, indicating that these proteases play a role in *Plasmodium* mosquito midgut invasion. Failure to generate genetic knockouts of these genes limited determination of the precise role of these proteases in parasite transmission but suggests that they are essential during the intraerythrocytic life cycle.

**Conclusions:**

PfPM VII and PfPM X are present in the mosquito-infective stages of *P. falciparum.* Standard membrane feeding assays demonstrate that antibodies against these proteins reduce the infectivity of *P. falciparum* for mosquitoes, suggesting their viability as transmission-blocking vaccine candidates. Further study of the role of these plasmepsins in *P. falciparum* biology is warranted.

**Electronic supplementary material:**

The online version of this article (doi:10.1186/s12936-016-1161-5) contains supplementary material, which is available to authorized users.

## Background

Despite reduced incidence and mortality rates malaria continues to have a significant global impact with more than 200 million people infected and a reported 438,000 deaths in 2015 alone [1]. Malaria is initiated when sporozoites are injected into a human from the bite of a *Plasmodium*-infected anopheline mosquito, with severe disease most often due to *Plasmodium falciparum*. Increasing resistance to common anti-malarial drugs and the lack of an effective vaccine enhance the importance of malaria as a global scourge requiring new approaches, including novel vaccine approaches to simultaneously target multiple parasite stages [2]. In addition to sporozoite-targeting malaria vaccine approaches that aim to prevent initial human infection, complementary lines of malaria vaccine research have also focused on so-called transmission-blocking vaccines or, as newly articulated “vaccines that interrupt malaria transmission” [2], which aim to prevent humans with *Plasmodium* gametocytaemia from infecting the mosquito vector [3, 4]. Transmission-blocking vaccines target proteins expressed on or secreted by sexual stage parasites, which develop in the mosquito midgut [5].

*Anopheles* species mosquitoes are the definitive hosts of *Plasmodium* parasites, which obligatorily complete development from sexual stages to sporozoites within the mosquito before transmission to humans. Upon ingestion by the mosquito, environmental changes encountered in the midgut stimulate mature gametocyte emergence from infected erythrocytes, in a process known as gametogenesis [6, 7]. Sexually dimorphic gametes fuse to generate zygotes in the midgut lumen. Zygotes then undergo sexual recombination and meiotic replication followed by transformation into polarized, motile ookinetes [8–10]. Ookinetes penetrate the midgut epithelium and form oocysts on the basal lamina. One ookinete that penetrates the midgut wall to form an oocyst has the potential to generate thousands of sporozoites, the form of the parasite that infects humans [11, 12]. Ookinete invasion of mosquito midgut is an important process for malaria transmission, but little is known about the molecular mechanisms involved.

Ookinetes produce stage-specific proteins important for subsequent midgut invasion, such as chitinase [13, 14], circumsporozoite- and thrombospondin-related adhesive protein [TRAP]-related protein (CTRP) [15, 16], von Willebrand A domain-related protein (WARP) [17, 18], *Plasmodium* 25/28 zygote/ookinete surface proteins (P25/28) [9, 19, 20], secreted ookinete adhesive protein (SOAP) [16, 21], and membrane attack perforin (MAOP) [22] amongst others. Because proteases play important roles during parasite infection of and development in the mosquito, they were considered as potential transmission-blocking vaccine targets [23–28]. Transcriptomic data suggested that *Plasmodium* aspartic proteases, known as plasmepsins, are expressed in sexual stage parasites [29]. The *P. falciparum* genome encodes ten *Plasmodium* aspartic proteases known as plasmepsins [30]. Plasmepsins I, II, IV and HAP are present in the asexual blood stage parasite food vacuole and are involved in haemoglobin degradation in the food vacuole of blood stage parasites [30–35]. In the endoplasmic reticulum of asexual blood stage parasites, Plasmepsin V processes proteins and directs export of effector proteins [36]. Plasmepsin VI plays an as-yet undefined but important role in parasite sporogonic development, particularly in early oocyst development in *Plasmodium berghei* [37]. Plasmepsin IV, in addition to its known role in haemoglobin degradation, is involved in *Plasmodium* ookinete invasion of the mosquito midgut [38].

Transcriptomic data have shown that *P. falciparum* plasmepsin VII (PfPM VII) mRNA is present in gametocytes, and plasmepsin X (PfPM X) mRNA is present during both in gametocytes and in zygotes and ookinetes [29]. The biological functions of PfPM VII and PfPMX remain unknown. Based on these observations, this study aimed to test the hypothesis that PfPM VII and PfPM X are targets of blocking mosquito midgut infection by *P. falciparum.* Such data would support the notion that these proteins would contribute to the interaction of the *P. falciparum* ookinete with the anopheline midgut and provide the basis for further development of these molecules as components of a malaria transmission-blocking vaccine.

## Methods

### Parasites and mosquitoes

*Plasmodium falciparum* strain NF54 from a master cell bank was used in this study, kindly provided under a material transfer agreement with Sanaria, Inc, Rockville, MD, USA. Parasites were maintained in asexual culture according to standard protocol [39]. Gametocytes and gametes were cultured in vitro according to the Ifediba and Vanderberg [40] modification of the Trager and Jensen method [41]; zygotes and ookinetes were cultured and purified as described [42–44].

*Anopheles gambiae* and *Anopheles stephensi* used in this study were a generous gift from Dr. Anthony James (University of California Irvine, Irvine, CA USA). Mosquitoes were maintained in an enclosed insectary at 26 °C and 80 % humidity with an automated 12-h light–dark cycle according to standard CDC protocol [45]. Mosquitoes used in this study were closely monitored and controlled according to the protocol for non-vertebrate animal subjects approved by the UCSD Institutional Animal Care and Use Committee (IACUC).

### DNA/RNA isolation and RT-PCR

*Plasmodium* parasites were either generated in vitro or isolated ex vivo from infected mosquitoes. For ex vivo-isolated mosquito-stage parasite samples, midguts from mosquitoes were dissected and homogenized; five midguts were pooled per sample at 24 h post-engorgement. Genomic DNA was isolated using NucleoSpin Blood (Macherey–Nagel, Bethlehem, PA, USA). Total RNA was isolated using RNeasy (Qiagen, Valencia, CA, USA) and contaminating DNA was removed using DNA-free (Ambion, Austin, TX, USA) according to manufacturer’s instructions. Reverse transcription was completed using gene-specific primers for PfPM VII, PfPM X and Pfs25 with SuperScript III first-strand synthesis system (Invitrogen, Carlsbad, CA, USA) according to manufacturer’s instructions. PCR on resulting cDNA was done using Platinum PCR SuperMix High Fidelity (Invitrogen, Carlsbad, CA, USA) with 250 nM of the same gene-specific primers for: Pfs25 (Fwd 5′-tgcgaaagttaccgtggatactg-3′; Rev 5′-tgcgaaagttaccgtggatactg-3′), PfPM VII (Fwd 5′-gcgccatgggtaaaaatgaagaattcacgaatccttattcc-3′, Rev 5′-gcgctcgagccttaaggttacatttcttttacttctaac-3′) and PfPM X (Fwd 5′- gtgatgaagaaagttacgttatatttgacacagg-3′; Rev 5′-gctcttgctactccaaccatagaagg-3′). Thirty-five cycles were run with an annealing temperature of 55 °C and an extension temperature of 68 °C.

### Production of recombinant PfPM VII and PfPM X in *Escherichia coli*

PfPM VII and PfPM X genes were amplified from genomic NF54 DNA to generate a gene that lacked the signal peptide. PCR products were gel purified on a 0.8 % agarose gel using the PureLink gel extraction kit (Invitrogen, Carlsbad, CA, USA), ligated into pCR4-TOPO (Invitrogen, Carlsbad, CA, USA), transformed into Top10 competent cells (Invitrogen, Carlsbad, CA, USA) and sequence-verified (Eton Bioscience, San Diego, CA, USA). PfPM VII and PfPM X genes were then cloned into expression vectors pET32 and pGEX 4T-1 (GE Healthcare, Piscataway, NJ, USA), respectively.

The recombinant PfPM VII-HIS-tagged (rPfPM VII-HIS) and PfPM X-GST-tagged (rPfPM X-GST) fusion proteins were expressed in Rosetta (Merck/Novagen, Darmstadt, Germany) competent cells according to standard protocol [46]. Briefly, competent cells were transformed with 250–500 ng of purified plasmid DNA, streaked on LB agar plates embedded with 100 µg/ml ampicillin and allowed to grow overnight at 37 °C. Fresh colonies were inoculated and grown to OD_600_ 0.5–1. Protein expression was then induced with 0.3 mM isopropyl β-D-1-thiogalactopyranoside (IPTG) for 0.5–18 h at 18 °C.

For rPfPM VII and rPfPM X protein verification, inclusion bodies were purified using BugBuster extraction reagent (Merck/Novagen, Darmstadt, Germany) and run on a 4–20 % Tris–Glycine SDS-PAGE gel that was then stained with Coomassie blue. A stained SDS-PAGE gel slice consistent with the predicted size of rPfPM VII-HIS and rPfPM X-GST fusion protein was excised and processed for mass spectrometry analysis (The Scripps Research Institute, La Jolla, CA, USA). The gel slices were destained and proteins were reduced with 10 mM DTT, alkylated with 55 mM iodoacetamide and digested in-gel with trypsin as previously described [47, 48]. Samples were analysed by mass spectrometry, and identified peptides were searched by BLAST against the *P. falciparum* genome.

### Production of monoclonal antibodies against PfPM VII and PfPM X

**Fig. 1:**
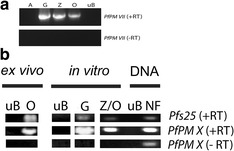
Plasmepsin VII and Plasmepsin X mRNA was detected in *Plasmodium falciparum* sexual stage parasites. **a** Total RNA isolated from in vitro-cultivated asexual stages (A), gametocytes (G), zygotes (Z), ookinetes (O), and uninfected human erythrocytes (uB). Samples were reverse transcribed and amplified using primers specific for PfPM VII (+RT). Samples that were not reverse transcribed (−RT) and amplified with PfPM VII-specific primers did not generate PCR product. **b** Total RNA isolated ex vivo from ookinete-containing mosquito midguts (O) or uninfected human blood (uB), in vitro-cultivated gametocytes (G) and mixed zygotes and ookinetes (Z/O), as well as DNA from *P. falciparum* (NF) was isolated. Samples were reverse transcribed and amplified using primers specific for PfPM X and pfs25 (+RT). Samples that were not reverse transcribed (−RT) and amplified with PfPM X-specific primers did not generate PCR product

PfPM VII peptide sequences EEFTNPYSIRKKDI, IQPDEQSEEDNVDG, and KIGFVRSKRNVTLR were derived from predicted immunogenic regions of the PfPM VII protein sequence using resources freely available from the Immune Epitope Database (iedb.org). PfPM X peptide sequences KKLQKHHESLKLGDVKYYV, VRNQTFGLVESES, and LKESYWEVKLD were derived from predicted immunogenic regions of the PfPM X protein sequence (Fig. 1). These synthetic peptides were created with an N-terminal cysteine to facilitate coupling to bovine serum albumin (BSA) as the antigenic carrier protein. Peptide composition was confirmed by mass spectrometry, and HPLC-purified peptides were used to immunize mice. The resulting hybridoma supernatants were screened against three sets of BSA-conjugated synthetic peptides, the same used to immunize mice, by enzyme linked immunosorbent assay (ELISA; A&G Pharmaceuticals, Columbia, MD, USA). Secondary screening of ELISA-positive supernatant was performed by western immunoblot assay against full-length rPfPM VII-HIS or rPfPM X-GST. Hybridoma lines generating mAb that screened positive in both ELISA and Western immunoblot assays were grown in Dulbecco’s modified eagle medium (DMEM, CellGro, Herndon, VA, USA) supplemented with 10 % fetal calf serum. Antibodies were either concentrated from hybridoma supernatant or purified mAb in PBS (A&G Pharmaceuticals, Columbia, MD, USA).

**Fig. 2:**
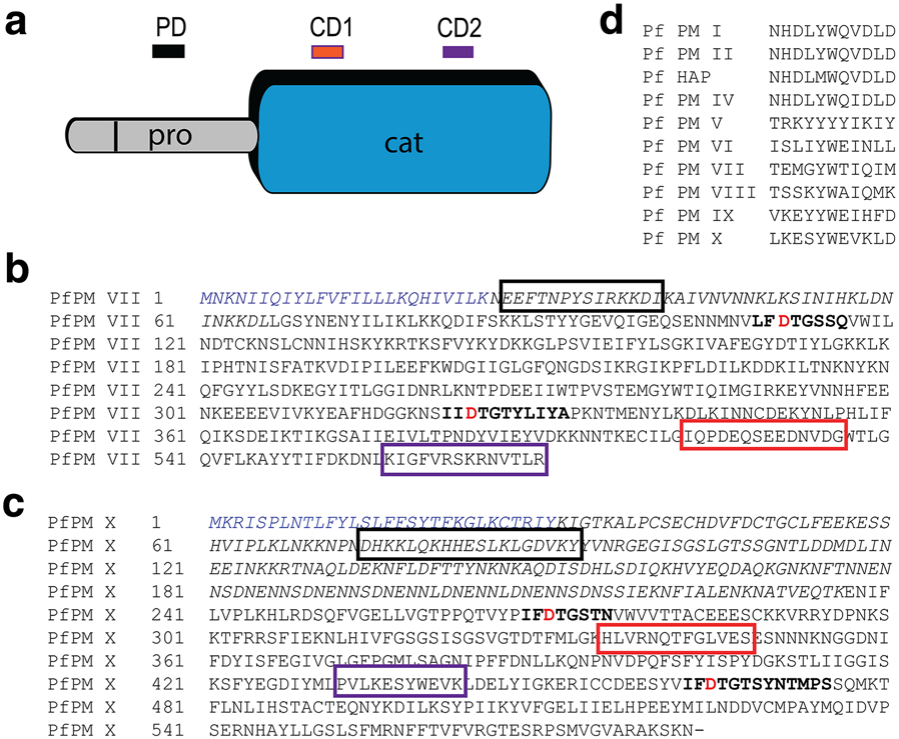
Peptide monoclonal antibodies directed against *Plasmodium falciparum* Plasmepsin VII and Plasmepsin X. **a** Schematic representation of Plasmepsin showing the predicted signal peptide (*blank*), pro-enzyme domains (*pro*), and catalytic domain (*cat*). Peptide monoclonal antibodies designed against three regions of PfPM VII and PfPM X are designated prodomain (PD, *black*), catalytic domain 1 (CD1, *red*) and catalytic domain 2 (CD2, *purple*). **b** Protein sequence of PfPM VII and **c**. PfPM X showing the predicted signal peptide (*blue*) and the predicted pro-enzyme domain (*italics*); the two active aspartic acid residues (*red*) are found within conserved regions (*bold*). Monoclonal antibody targets to prodomain (*black*), CD1 (*red*), and CD 2 (*purple*). **d** Alignment of 3G6 peptide target between all *P. falciparum* plasmepsins shows moderate conservation between PfPM X and PfPM IX at this site

After screening against rPfPM VII, two lines, 1B4 and 2B1, generated antibodies directed against each catalytic domain of PfPM VII. After screening against rPfPM X, one line, 6A9, generated antibody directed against the pro-enzyme domain, and one line, 3G6, generated antibody directed against the catalytic domain (Fig. 2). mAb 6A9 was directed against a peptide region unique to PfPM X while 3G6 is directed against a peptide region that is moderately conserved with PfPM IX (Fig. 2).

### Immunofluorescence assay

Fixed, permeabilized *P. falciparum* gametocytes, gametes, zygotes and ookinetes were analysed by IFA. Parasites were fixed on glass slides with 100 % acetone at −20 °C for at least 20 min and then rehydrated by two changes of PBS for 5 min each at room temperature. For membrane permeabilization and blocking of nonspecific binding, fixed cells were incubated in PBS supplemented with 3 % bovine serum albumin and 0.1 % Triton X-100 for 1 h at room temperature. The preparations were then incubated with mAb (1:1000 dilution) for 1 h at room temperature followed by either FITC-conjugated anti-mouse IgG or Alexa Fluor 488 rabbit anti-mouse IgG (1:200 dilution) (Molecular Probes, Invitrogen, USA). Nuclei were visualized with 300 nM DAPI (4, 6-diamidino-2-phenylindole) (Pierce Biotechnology, Rockford, IL, USA). Slides were washed an additional six times in Tris-buffered saline (TBS) for a total of 30 min then mounted with coverslips using Dako mounting medium (Dako, Carpinteria, USA). Preparations were examined by deconvolution microscopy using an Olympus BX51 fluorescence microscope and Olympus DP71 camera (Olympus, Center Valley, CA, USA).

### Immunoelectron microscopy localization of PfPM VII and PfPM X

Cells were fixed with 2.5 % glutaraldehyde in 0.1 M cacodylate buffer for 2 h at room temperature, postfixed in 1 % OsO_4_ in 0.1 M cacodylate buffer (1 h) at room temperature, and embedded in LX-112 (Ladd Research, Williston, VT), as described previously [49]. Cryosections were made, applied to grids, blocked in 1 % BSA in PBS for 1 h, incubated in 1:500 dilution of mAb against PfPM VII, PfPM X or IgG isotype control, washed and incubated with anti-mouse IgG conjugated to 5 nm colloidal gold particles as described previously [50]. Stained sections were examined using a Philips CM-10 electron microscope.

### Western immunoblot analysis

*Plasmodium falciparum* parasites were purified, pelleted and resuspended in 250 µl of lysis buffer (4 M urea, 0.4 % Triton X-100, 50 mM Tris, 5 mM EDTA, 10 mM MgSO_4_, pH 8.0) supplemented with Complete protease inhibitor cocktail (Roche Applied Sciences, Indianapolis, IN, USA). Parasites were processed by three cycles of freeze–thaw lysis followed by sonication on ice for 5 min in 30 s bursts using a Misonix Sonicator 3000 with an output setting of 7 (Misonix, Farmingdale, NY, USA). Protein concentrations were determined by BCA assay (Bio-Rad, Hercules, CA, USA). 100 µg of each sample was mixed with Laemmli SDS-loading buffer (160 mM Tris, 10 % SDS, 20 % glycerol, 5 % 2-mercaptoethanol, 0.01 % bromophenol blue, pH 8.0) and boiled for 10 min. Proteins were separated on Novex 10–20 % SDS-PAGE mini-gels (Invitrogen, Carlsbad, CA 92008 USA) and transferred to nitrocellulose membranes. Membranes were blocked in TBS/5 % non-fat milk/0.05 % Tween-20, pH 8.0 for 1 h. Blots were probed with primary antibody diluted 1:2000 in blocking buffer for 1 h. Following six 10-min washes in blocking buffer, blots were probed with peroxidase-conjugated anti-mouse secondary antibody diluted 1:20,000 in blocking buffer. Blots were washed six times in blocking buffer, twice in TBS and then developed with chemiluminescent substrate (KPL, Gaithersburg, MD, USA).

### Membrane feeding assays

Standard membrane feeding assays (SMFA) were performed to determine the ability of antibodies against Pf PMVII and PfPMX to affect infectivity of *P. falciparum* for mosquitoes. One day prior to the assay, female *An. stephensi* or *An. gambiae* mosquitoes aged 3–7 days post-emergence were segregated into cartons of 40–60 mosquitoes, and starved overnight. Mature *P. falciparum* gametocytes were examined for their ability to exflagellate and only those cultures with at least 10 exflagellating centres per 40X field were used for SMFA. Cultures with mature gametocytes were mixed with fresh human serum and red blood cells, plus antibodies, then fed to *An. stephensi or An. gambiae* using water-jacketed glass membrane feeders as previously described [14]. Isotype immunoglobulin G2b (IgG2b) was used as a negative control. Twenty minutes after the start of the feed, membrane feeders were disengaged and non-engorged mosquitoes were removed from cartons. *P. falciparum*-infected mosquitoes were kept in a secured incubator separate from non-infected mosquitoes. Infected mosquitoes were fed with 8 % fructose/0.05 % p-aminobenzoic acid in sterile water ad libitum and maintained at 26–28 °C and 80 % relative humidity [51, 52].

On day 8–10 post blood meal, mosquito midguts were dissected, stained with mercurochrome and examined with a light microscope for the presence of oocysts [52]. All manipulations were done in accordance with UCSD IACUC-approved protocol for non-vertebrate research animals. Differences in infection rate and geometric means between test mAb and negative control groups were assessed using the non-parametric Mann–Whitney U test. Samples were considered to be statistically significant at a *p* value ≤0.01.

## Results

### PfPM VII and PfPM X mRNA was transcribed in *P. falciparum* sexual stages

RT-PCR of RNA isolated from *P. falciparum* sexual stage parasites generated in vitro and dissected from mosquitoes ex vivo demonstrated that PfPM VII and PfPM X mRNA was detected in gametocytes and mixed zygotes/ookinetes (Fig. 1). Conventional PCR of RNA samples not treated with reverse transcriptase did not produce PCR product and demonstrated that RNA samples were DNA free (Fig. 1).

### PfPM VII and PfPM X protein expression in sexual stage parasites

**Fig. 3:**
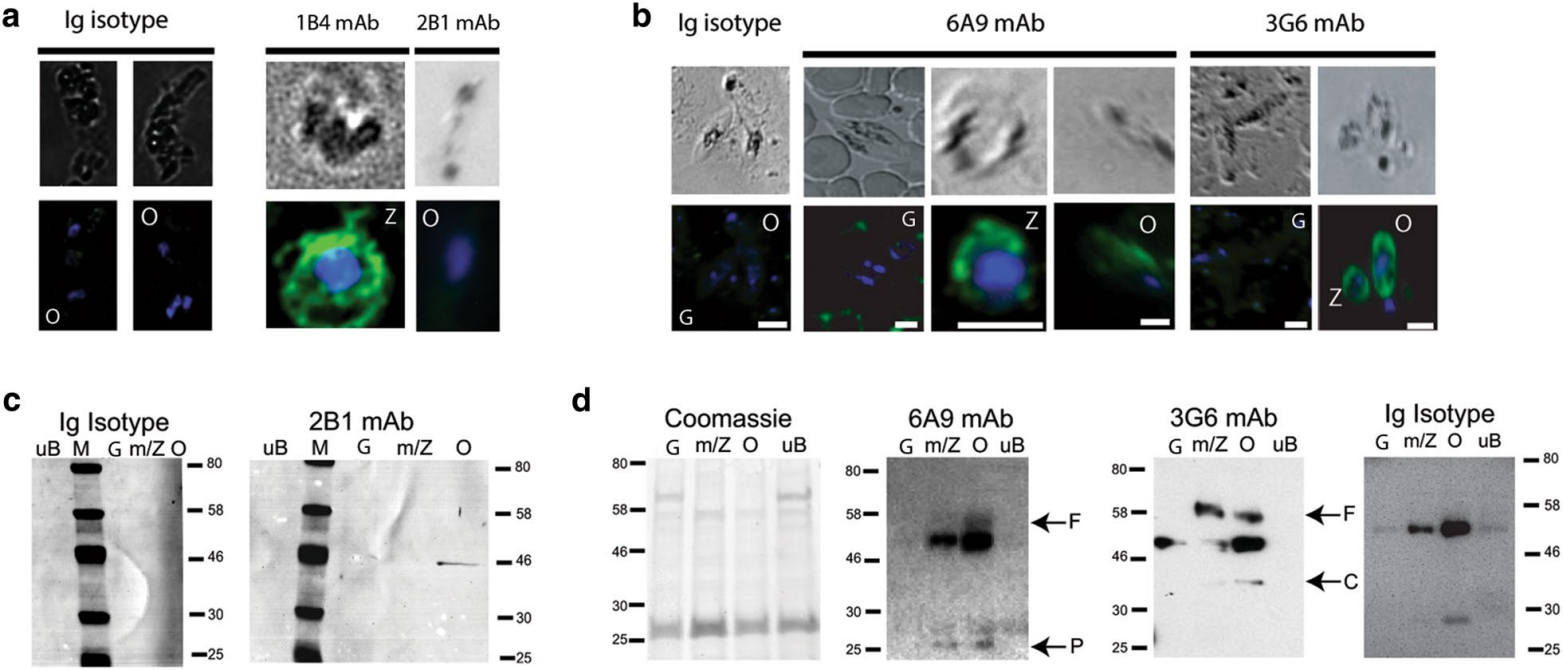
PfPM VII and PfPM X expression in *Plasmodium falciparum* sexual stage parasites demonstrated by IFA and western immunoblot. IFA of in vitro-cultivated parasites was performted using primary antibodies 1B4, 2B1, 6A9, 3G6 or isotype IgG2b and subsequently labelled with Alexa Fluor 488-labelled anti-mouse antibodies (*green*); nuclei are visualized with DAPI (*blue*). **a** IFA of in vitro-cultivated *P. falciparum* demonstrated PfPM VII expression in zygotes (Z) and ookinetes (O). **b** IFA of in vitro-cultivated *P. falciparum* demonstrated PfPM X expression in zygotes (Z) and ookinetes (O) but not gametocytes (G). **c** Western immunoblot analysis of total protein isolated from *P. falciparum* sexual stage parasites. Antibodies directed against PfPM VII recognized a ~46 kDa protein expressed in ookinetes (O) but not gametocytes (G). This protein is between the predicted sizes of full length PfPM VII at 52 kDa and the catalytic domain at 43 kDa. **d** Western immunoblot analysis of total protein isolated from *P. falciparum* sexual stage parasites. Both 6A9 and 3G6 recognized protein expressed in mixed gametes and zygotes (m/Z) and ookinetes (O) but not gametocytes (G). Bands are consistent with the predicted sizes of full length PfPM X at 61 kDa (←F), the PfPM X catalytic domain at 36 kDa (←C), and the PfPM X proenzyme domain at 23 kDa (←P). IgG isotype control did not recognize the 61 kDa or 23 kDa bands on western immunoblot but continued to recognize the bands at 42–55 kDa and 27–30 kDa

IFA of *P. falciparum* sexual stage parasites using mAbs directed against PfPM VII and PfPM X demonstrated diffuse, cytoplasmic localization in *P. falciparum* zygotes and ookinetes but not gametocytes (Fig. 3). Neither PfPM VII nor PfPM X were found to be localized within specific sub-cellular compartments, such as micronemes or endoplasmic reticulum, or the ookinete cell surface as determined by immunoelectron microscopy.

Western immunoblot confirmed IFA results demonstrating PfPM VII and PfPM X protein expression in sexual stage parasites. Antibody 2B1 recognized a single ~ 46 kDa band in ookinetes but no other sexual stage parasite; this protein is close to the predicted 52 kDa size of the full length protein and the 42 kDa size of the PfPM VII catalytic domain (Fig. 3). Antibodies 6A9 and 3G6 recognized multiple bands in mixed gamete/zygote samples and ookinete samples, but not gametocyte samples. Antibody 6A9 recognized two bands: a 56–60 kDa protein, consistent with the predicted 61 kDa size of full length PfPM X, and a faint 17–25 kDa protein, consistent with the predicted 23 kDa size of the PfPM X pro-enzyme domain. Antibody 3G6 also recognized a 56–60 kDa protein in addition to a faint 32–40 kDa protein, consistent with the predicted 36 kDa size of the catalytic domain. Of note, 6A9 and 3G6 recognized two bands in parasite lysate, one at 42–55 kDa and the second at 27–30 kDa; these bands were also recognized by IgG isotype control antibody, indicating that these protein bands are non-specific (Fig. 3).

### Antibodies directed against PfPM VII or PfPM X moderately decreased *Plasmodium falciparum* transmission to *Anopheles* in SMFAs

To determine whether antibodies against either PfPM VII or PfPM X could affect *P. falciparum* transmission to mosquitoes, female *An. stephensi* and *An. gambiae* were fed infectious gametocytes mixed with 1B4, 2B1, 6A9, 3G6 or isotype IgG negative control antibodies, respectively, in SMFAs (Table 1, 2). Mosquito midguts were dissected 8–10 days post-blood meal to determine prevalence and intensity of infection.

**Table 1:** Effect of mAbs against PfPM VII on *Plasmodium falciparum* infectivity to *Anopheles stephensi* mosquitoes

Exp	Group	Intensity (geometric mean oocysts/mosquito)	Oocyst range	Prevalence (no. infected/total engorged mosquitoes)
1	200 μg/ml IgG negative	2.28	0–10	31/39 (79.5 %)
	200 μg/ml 1B4	0.07*	0–2	3/37 (8.1 %)
	400 μg/ml IgG negative	22.18	7–58	24/24 (100 %)
	400 ug/ml 2B1	7.7*	0–54	13/17 (76.5 %)
2	200 μg/ml IgG negative	24.23	0–121	34/40 (85.0 %)
	200 μg/ml 1B4	3.47*	0–45	20/40 (50.0 %)
	400 μg/ml IgG negative	9.15	0–88	29/38 (76.3 %)
	400 μg/ml 2B1	3.17*	0–81	20/33 (66.6 %)
3	200 μg/ml IgG negative	12.67	0–55	35/40 (87.5 %)
	200 μg/ml 1B4	2.41*	0–29	8/19 (42.1 %)
	400 μg/ml IgG negative	14.74	0–68	31/32 (96.9 %)
	400 μg/ml 2B1	6.5*	0–65	29/34 (85.3 %)

mAbs and IgG isotype control data from three independent SMFAs. Transmission-blocking activities measured as effects on intensity and prevalence.

* Statistical significance determined by Mann–Whitney U test, control versus mAb, *p* value ≤0.01

**Table 2:** Effect of mAbs against PfPM X on *Plasmodium falciparum* infectivity to *Anopheles gambiae* mosquitoes

Group	Intensity (geometric mean oocysts/mosquito)	Oocyst range	Prevalence (no. infected/total engorged mosquitoes)
200 μg/ml IgG negative	2.6	0–55	62/80 (77.5 %)
100 μg/ml 3G6	1.7	0–108	48/80 (60.0 %)
200 μg/ml 3G6	1.5*	0–148	34/76 (44.7 %)
200 μg/ml 6A9	2.4	0–59	33/51 (64.7 %)

mAbs and IgG isotype control data from triplicate SMFAs under the same conditions, data combined. Transmission-blocking activities measured as effects on intensity and prevalence

* Statistical significance determined by Mann–Whitney U test, control versus mAb, *p* value ≤0.01

The presence of antibodies directed against either PfPM VII or PfPM X in an infectious blood meal significantly decreased *P. falciparum* transmission to mosquitoes at high antibody concentrations (Tables 1, 2). Mosquitoes fed an infectious bloodmeal with 200 µg/ml of 1B4 or 400 µg/ml of 2B1 had a 35–71.4 or 9.7–23.5 % reduction in prevalence compared to groups fed with IgG isotype control antibody (Table 1). Additionally, oocyst intensity of infected mosquitoes fed with1B4 or 2B1 was reduced by 81–97 or 56–65 % compared to IgG isotype control (*p* value < 0.01) (Table 1). Mosquitoes fed an infectious blood meal with 100 or 200 µg/ml of 3G6 had a 18 or 33 % reduction in prevalence compared to IgG isotype control (Table 2). Additionally, the oocyst intensity of infected mosquitoes fed with 100 μg/ml of 3G6 was reduced by 35 % compared to IgG isotype control, the oocyst intensity of infected mosquitoes fed with 200 μg/ml of 3G6 was reduced by 42 % compared to IgG isotype controls (*p* value < 0.01) (Table 2). The presence of 200 µg/ml of 6A9 in an infectious bloodmeal only reduced *P. falciparum* transmission to mosquitoes by 13 %, and oocyst intensity was comparable to IgG isotype controls (Table 2).

Further attempts were made to elucidate the precise role of PfPM VII and PfPM X during *P. falciparum* sexual development and transmission to mosquitoes. Unfortunately, we were unable to produce active, recombinant protease in either an *E. coli*-based expression system or a cell-free wheat germ expression system. The generation of PfPM VII and PfPM X knockout parasites were unsuccessful (Additional files 1, 2).

## Discussion

The data reported here indicate that that PfPM VII and PfPM X are expressed in ookinetes and contribute to *P. falciparum* transmission to *Anopheles* mosquitoes. This is the first observation that these plasmepsins play an important role in *Plasmodium* infection of mosquitoes. Transcriptomic data from all the life cycle stages of *P. falciparum* demonstrate mRNA expression of PfPM VII and PfPM X in sexual stage forms. These previously published microarray data were confirmed by RT-PCR on in vitro-cultivated gametocytes, gametes, zygotes and ookinetes and ex vivo-harvested zygotes and ookinetes. Anti-peptide monoclonal antibodies directed against PfPM VII or PfPM X demonstated that these proteins are expressed in sexual stage parasite forms. Interestingly, PfPM VII and PfPM X mRNAs are expressed in *P. falciparum* gametocytes, but protein was not detected until zygote and gamete development, respectively. This inconsistency between mRNA and protein expression was not explored in this work, but this pattern suggests that PfPM VII and PfPM X protein expression may be regulated by translational repression, a mechanism used to regulate other *Plasmodium* sexual stage proteins [53–58]. Similar observations have been seen with Pfs25 and chitinase, whose mRNAs but not proteins have been detected in gametocytes [50, 59].

Another mechanism to regulate protease activity is zymogen processing. Plasmepsins are known to harbour a proenzyme domain that functions to maintain the enzyme in a catalytically inactive state. Plasmepsins only become catalytically active when the proenzyme domain is cleaved and separated from the catalytic domain. Both PfPM VII and PfPM X contain predicted proenzyme domains. Western immunoblot of all three mAbs targeting PfPM VII only detected one protein in parasite lysate; this finding was unexpected as PfPM VII also contains a predicted proenzyme domain. Additionally, the protein recognized is smaller than the predicted size of the full-length enzyme but large than the predicted size of the PfPM VII catalytic domain. Although we would expect to see two bands corresponding to the full-length protein and the proenzyme domain alone, it is possible that PfPM VII is not processed and activated in the ookinete. If PfPM VII is secreted or cell-surface associated, a hypothesis not currently supported by IFA or immunoelectron data, it is possible that proenzyme domain is cleaved outside of the parasite. Given this result, it is likely that the full-length protein is processed at a site upstream of the mAb target region but that the proenzyme domain has not yet been cleaved in the ookinete, resulting in a smaller-than expected full-length protein. Western immunoblot analysis demonstrated that 6A9, which targets the PfPM X proenzyme domain, recognized proteins consistent with the sizes of the full-length enzyme and the cleaved pro-enzyme domain while 3G6, which targets the catalytic domain, recognized proteins consistent with the sizes of the full-length enzyme and the cleaved catalytic domain. This observation suggests that PfPM X is processed, and potentially catalytically active, during *P. falciparum* sexual stage development. Western immunoblot demonstrate that both PfPM VII and PfPM X are processed, and that PfPM X is likely catalytically active in the ookinete.

The majority of proteins that are both important for *Plasmodium* transmission to mosquitoes and good targets for transmission-blocking vaccines are either localized to the parasite cell surface or secreted from the ookinete. Cell-surface proteins, such as P25/P28, mediate parasite-host interactions and may be essential components of signalling-pathways important for ookinete motility [15, 19, 21, 60]. Similarly, ookinete-secreted proteins and enzymes, such as chitinase, CTRP and WARP, modify the mosquito midgut environment to allow *Plasmodium* invasion and infection of the mosquito midgut [13, 14, 17, 18]. Neither PfPM VII nor PfPM X localized to the parasite cell surface or micronemes. IFA and immunoelectron microscopy demonstrated diffuse cytoplasmic localization of mAbs directed against PfPM VII and PfPM X.

Previous studies showed that pepstatin A, an aspartic protease-specific inhibitor, ALLN and ALLM, two plasmepsin convertase inhibitors, interrupted *Plasmodium* transmission to mosquitoes [38]. The presence of antibodies directed against PfPM VII or PfPM X significantly reduced *P. falciparum* infection of *Anopheles*. However, relatively high concentrations of 1B4, 2B1 and 3G6 were required to achieve an effect. It is possible that the antibodies had low affinity, resulting in the low level of interruption of the transmission. It is also possible that these Plasmepsins were important, but not essential, for *P. falciparum* transmission to *Anopheles* mosquitoes, consistent with previous reports with a *P. berghei* Plasmepsin VII knockout mutant [61]. Equally, if not more likely is the possibility that loss of PfPM VII or PfPM X function in the presence of mAb was compensated for by the action of related proteases, such as Plasmepsin IX, which are similarly upregulated in sexual stage parasite forms [29]. The equivocal result seen in SMFAs using 6A9, directed against the PfPM X proenzyme domain, is expected if the proenzyme domain of PfPM X is cleaved and no longer associated with the activated catalytic domain in ookinetes. These data indicate that there is still much to learn about the role of plasmepsins in *Plasmodium* transmission to mosquitoes. Further work is needed to explore these interesting possibilities.

## Conclusions

Knowledge of the *Plasmodium* sexual cycle and the molecular mechanisms underlying ookinete invasion of the mosquito midgut is essential for understanding parasite developmental programmes and parasite-mosquito interactions. In this study, we have investigated the expression and functional significance of PfPM VII and PfPM X in malaria transmission to mosquitoes. We demonstrated that these proteins are expressed in *P. falciparum* ookinetes and that antibodies directed against these proteins reduced parasite transmission to *Anopheles* mosquitoes when used at high concentration in mosquito infection experiments. This observation suggests that PfPM VII and PfPM X facilitate *Plasmodium* infection of mosquitoes. This data complements discoveries demonstrating that *P. gallinaceum* plasmepsin IV was important for parasite infection of the mosquito vector [38] and *P. berghei* plasmepsin VII was dispensable in its life cycle [61]. Together, these results suggest that plasmepsins expressed in the *Plasmodium* ookinete function in ways distinct from plasmepsins expressed in asexual parasites. These findings raise a number of interesting questions. What other plasmepsins are expressed in sexual stage parasites? If these plasmepsins are involved in midgut invasion or sexual stage parasite development, what pathways are involved? Further investigation of the role(s) of plasmepsins during sexual development and invasion of mosquitoes will likely lead to novel insights into *Plasmodium* biology and provide new targets for transmission-blocking vaccines.

## Additional files

10.1186/s12936-016-1161-5 Production of active rPfPM VII and rPfPM X could not be achieved using multiple expression systems.
10.1186/s12936-016-1161-5 PfPM VII and PfPM X appear to be essential for *P. falciparum* asexual development.

## Abbreviations

PfPM VII: *Plasmodium falciparum* plasmepsin VII
PfPM X: *Plasmodium falciparum* plasmepsin X
RT-PCR: reverse transcriptase polymerase chain reaction
SMFA: standard membrane feeding assay
Pfs25: *Plasmodium falciparum* 25 kDa surface molecule
IFA: immunofluorescence assay
ELISA: enzyme-linked immunosorbent assay
mAb: monoclonal antibody
