# Supplementary material for: Plasmodium falciparum ookinete expression of plasmepsin VII and plasmepsin X
Source: Malar J. 2016 Feb 24;15:111. doi: 10.1186/s12936-016-1161-5 (PMC4765185; doi:10.1186/s12936-016-1161-5)
Supplement: Supplementary file 1 — 10.1186/s12936-016-1161-5 Production of active rPfPM VII and rPfPM X could not be achieved using multiple expression systems. [file 12936_2016_1161_MOESM1_ESM.docx]

### Additional file 1 – Production of active rPfPM VII and rPfPM X could not be achieved using multiple expression systems

Generation of active, rPfPM VII and rPfPM X was attempted using multiple expression systems and multiple expression protocols, none of which resulted in active protease. The sequences encoding the post-signal peptide pro-plasmepsin of PfPM VII and PfPM X were cloned into pET32 and pGEX 4T-1 expression vectors to generate pET32-PfPM VII and pGEX 4T-1-PfPM X. Under all expression conditions tested, the majority of rPfPM VII-His and rPfPM X-GST was produced as inclusion bodies, although a small fraction of rPfPM VII-His or rPfPM X-GST could be purified from the soluble fraction using Ni-NTA agarose or GST-conjugated sepharose beads. The purified soluble fraction was dialyzed and concentrated.

Inclusion bodies were isolated, solubilised and refolded using protocols previously shown to be successful for PM II and PM IV [1-6]. Briefly, inclusion bodies were purified and solubilised in both 4-8 M urea and 4-6 M guanidine hydrochloride overnight. Solubilised protein was then refolded in Tris buffer, pH 7 – 10 in the presence or absence of dithiothreitol (DTT), 2-mercaptoethanol, arginine, glutathione, ethylenediaminetetraacetic acid (EDTA), polyethylene glycol (PEG) MgCl2, and/or CaCl2. Refolded proteins were visually scanned for the presence of precipitates. Conditions previously defined (modified from [2, 3]) did not result in rPfPM VII or rPfPM X protein precipitation and were used to solubilise and refold recombinant protein for activity assays. Refolded protein was dialyzed with Slide-A-Lyzer Dialysis cassettes (Thermo Fisher Scientific, Waltham, MA, USA) with 4 buffer changes and concentrated using Centricon columns (Millipore Corp., Billerica, MA, USA). Both concentrated soluble and purified, refolded inclusion body fractions were assayed for activity using the Pierce fluorometric Quanticleave kit (Thermo Fisher Scientific, Waltham, MA, USA) according to manufacturer’s instructions. The resulting activity in refolded fractions, though greater than activity from negative control samples, was below the threshold activity of trypsin positive control (data not shown).

To ensure that the 25-27 kDa GST tag on the rPfPM X-GST fusion protein did not block the catalytic active site, refolded and soluble protein was cleaved with thrombin to remove the GST tag. Thrombin-cleaved rPfPM X-GST failed to exhibit significant activity compared to control. The pro-enzyme domain is known to be important for aspartic protease processing and proper protein folding and retention of this domain may prevent protease activation [7]. The acidic food vacuole plasmepsins, PM II and PM IV, exhibit autocatalytic activity in their native, low pH environment [8], resulting in cleavage of the proenzyme domain. A range of pH refolding buffers from pH 2.0 to 10.0 were tested to optimize recombinant protein refolding, but did not result in active rPfPM VII or rPfPM X. To ensure that the pro-enzyme domain did not interfere with activity, an enterokinase cleavage site was engineered at the predicted PfPM X pro-domain cleavage site. Cleavage of the resulting protein with enterokinase did not result in active rPfPM X (data not shown).

The production of recombinant, active *Plasmodium* enzymes in *E. coli* has been typically fraught with difficulty, in part because of the AT-rich *P. falciparum* genome. *E. coli*-codon optimized genes [9, 10] or use of a cell-free wheat germ transcription and translation system [11, 12] have improved production of properly folded recombinant protein. To improve production of soluble rPfPM X-GST, the gene encoding PfPM X was *E. coli*-codon optimized. The *E. coli*-codon optimized PM X gene was expressed in Rosetta bacteria as well as SHuffle bacteria (New England Biolabs, Ipswich, MA, USA). Expression of *E. coli*-codon optimized genes in Rosetta or SHuffle did not significantly improve yields of soluble protein. Expression of rPfPM VII and rPfPM X using a cell-free wheat germ transcription and translation system did not generate active, recombinant PM X.

References

1. Gulnik SV, Afonina EI, Gustchina E, Yu B, Silva AM, Kim Y, Erickson JW: **Utility of (His)6 tag for purification and refolding of proplasmepsin-2 and mutants with altered activation properties.** *Protein Expr Purif* 2002, **24:**412-419.

2. Luker KE, Francis SE, Gluzman IY, Goldberg DE: **Kinetic analysis of plasmepsins I and II aspartic proteases of the Plasmodium falciparum digestive vacuole.** *Mol Biochem Parasitol* 1996, **79:**71-78.

3. Shenai BR, Sijwali PS, Singh A, Rosenthal PJ: **Characterization of native and recombinant falcipain-2, a principal trophozoite cysteine protease and essential hemoglobinase of Plasmodium falciparum.** *J Biol Chem* 2000, **275:**29000-29010.

4. Hill J, Tyas L, Phylip LH, Kay J, Dunn BM, Berry C: **High level expression and characterisation of Plasmepsin II, an aspartic proteinase from Plasmodium falciparum.** *FEBS Lett* 1994, **352:**155-158.

5. Wyatt DM, Berry C: **Activity and inhibition of plasmepsin IV, a new aspartic proteinase from the malaria parasite, Plasmodium falciparum.** *FEBS Lett* 2002, **513:**159-162.

6. Li F, Templeton TJ, Popov V, Comer JE, Tsuboi T, Torii M, Vinetz JM: **Plasmodium ookinete-secreted proteins secreted through a common micronemal pathway are targets of blocking malaria transmission.** *J Biol Chem* 2004, **279:**26635-26644.

7. Horimoto Y, Dee DR, Yada RY: **Multifunctional aspartic peptidase prosegments.** *N Biotechnol* 2009, **25:**318-324.

8. Kim YM, Lee MH, Piao TG, Lee JW, Kim JH, Lee S, Choi KM, Jiang JH, Kim TU, Park H: **Prodomain processing of recombinant plasmepsin II and IV, the aspartic proteases of Plasmodium falciparum, is auto- and trans-catalytic.** *J Biochem* 2006, **139:**189-195.

9. Yadava A, Ockenhouse CF: **Effect of codon optimization on expression levels of a functionally folded malaria vaccine candidate in prokaryotic and eukaryotic expression systems.** *Infect Immun* 2003, **71:**4961-4969.

10. Flick K, Ahuja S, Chene A, Bejarano MT, Chen Q: **Optimized expression of Plasmodium falciparum erythrocyte membrane protein 1 domains in Escherichia coli.** *Malar J* 2004, **3:**50.

11. Tsuboi T, Takeo S, Iriko H, Jin L, Tsuchimochi M, Matsuda S, Han ET, Otsuki H, Kaneko O, Sattabongkot J, et al: **Wheat germ cell-free system-based production of malaria proteins for discovery of novel vaccine candidates.** *Infect Immun* 2008, **76:**1702-1708.

12. Takeo S, Hisamori D, Matsuda S, Vinetz J, Sattabongkot J, Tsuboi T: **Enzymatic characterization of the Plasmodium vivax chitinase, a potential malaria transmission-blocking target.** *Parasitol Int* 2009, **58:**243-248.
