# Supplementary material for: Plasmodium falciparum ookinete expression of plasmepsin VII and plasmepsin X
Source: Malar J. 2016 Feb 24;15:111. doi: 10.1186/s12936-016-1161-5 (PMC4765185; doi:10.1186/s12936-016-1161-5)
Supplement: Supplementary file 2 — 10.1186/s12936-016-1161-5 PfPM VII and PfPM X appear to be essential for P. falciparum asexual development. [file 12936_2016_1161_MOESM2_ESM.docx]

### Additional file 2 – PfPM VII and PfPM X appear to be essential for *P. falciparum* asexual development

A single crossover recombination strategy was used to knock out the genes encoding PfPM VII and PfPM X. A 1 kb region of each gene was PCR amplified from *P. falciparum* genomic DNA, sub-cloned into pCR 2.1 (Invitrogen, Carlsbad, CA, USA), transformed into Top10 cells (Invitrogen, Carlsbad, CA, USA) and sequence-verified (Eton Biosciences, San Diego, CA, USA). Sequence-verified PfPM VII and PfPM X fragments were then cloned into pCAM-BSD [1] or pHDWT vector (Malaria Research and Reference Reagent Resource Center, Manassas, VA, USA deposited by Dr. D.A. Fidock and T.E. Wellems [2]) to generate pCAM-BSD-PfPM VII and pHDWT-PfPM X, respectively.

Asexual *P. falciparum* cultures were sorbitol-synchronized 48 hours prior to transfection. Ring stage parasites at 5-10% parasitemia were electroporated using the Bio-Rad Gene Pulser (Bio-Rad, Hercules, CA, USA) or Nucleofector technology (Lonza/Amaxa, Basel, Switzerland) using 100 µg or 10 µg of purified, endotoxin-free plasmid DNA resuspended in cytomix (120 mM KCl, 0.15 mM CaCl2, 5mM MgCl2, 2 mM ethylene glycol tetraacetic acid (EGTA), 10 mM K2HPO4/KH2PO4, 25 mM HEPES) or 100 µl T-cell solution (Lonza/Amaxa, Basel, Switzerland), respectively (Fidock and Wellems, 1997). The Gene Pulser II was set to 0.31 kV, 950 µF capacitance; the Amaxa Nucleofector was set to program U-33. Electroporated parasites were immediately transferred to warm, complete medium (RPMI1640, 25 mM HEPES, 2 mM L-glutamine, 2.4 g/L NaHCO3, 50 mg/L hypoxanthine, 10% heat-inactivated AB+ human serum).

Drug pressure was applied using either blastocidin at 2.5 ug/ml for pCAM-BSD-PfPM VII transfected parasites or the anti-folate drug WR99210 at 2.5 nM for pHDWT-PfPM X transfected parasites. Drug pressure was started 24-48 hours after transfection and continued daily; 50 µl of blood was added to cultures under selection every 3 days and cultures were split every 10 days until parasite replication rates maintained wild-type levels for one week [3]. Transfected parasites were then selected with on-off drug pressure: cycles of drug pressure for two weeks followed by two weeks without drug pressure. When parasite replication rates under drug selection (on) were equal to replication rates without drug selection (off), parasites were cloned by limiting dilution and maintained under drug pressure for an additional two weeks. DNA was extracted from cloned, transfected parasites for PCR and southern blot analysis of knockout vector integration.

Southern blot analysis was done to determine whether drug-resistant parasites contained genomically disrupted PfPM VII or PfPM X. For Southern blot, probes were labelled with digoxigenin (DIG) by PCR using the PCR DIG Probe synthesis kit (Roche Applied Sciences, Indianapolis, IN, USA) according to manufacturer’s instructions. Southern blot analysis was done using Southern blot kit (Roche Applied Sciences, Indianapolis, IN, USA) according to manufacturer’s instructions.

References

1. Sidhu AB, Valderramos SG, Fidock DA: **pfmdr1 mutations contribute to quinine resistance and enhance mefloquine and artemisinin sensitivity in Plasmodium falciparum.** *Mol Microbiol* 2005, **57:**913-926.

2. Fidock DA, Wellems TE: **Transformation with human dihydrofolate reductase renders malaria parasites insensitive to WR99210 but does not affect the intrinsic activity of proguanil.** *Proc Natl Acad Sci U S A* 1997, **94:**10931-10936.

3. Fidock DA, Nomura T, Wellems TE: **Cycloguanil and its parent compound proguanil demonstrate distinct activities against Plasmodium falciparum malaria parasites transformed with human dihydrofolate reductase.** *Mol Pharmacol* 1998, **54:**1140-1147.
